# Functional Recovery from Neural Stem/Progenitor Cell Transplantation Combined with Treadmill Training in Mice with Chronic Spinal Cord Injury

**DOI:** 10.1038/srep30898

**Published:** 2016-08-03

**Authors:** Syoichi Tashiro, Soraya Nishimura, Hiroki Iwai, Keiko Sugai, Liang Zhang, Munehisa Shinozaki, Akio Iwanami, Yoshiaki Toyama, Meigen Liu, Hideyuki Okano, Masaya Nakamura

**Affiliations:** 1 Department of Rehabilitation Medicine, Keio University School of Medicine, Tokyo, Japan; 2 Department of Orthopaedic Surgery, Keio University School of Medicine, Tokyo, Japan; 3 Department of Physiology, Keio University School of Medicine, Tokyo, Japan; 4 Department of Neuroscience, City College of the City University of New York, NY, USA

## Abstract

Most studies targeting chronic spinal cord injury (SCI) have concluded that neural stem/progenitor cell (NS/PC) transplantation exerts only a subclinical recovery; this in contrast to its remarkable effect on acute and subacute SCI. To determine whether the addition of rehabilitative intervention enhances the effect of NS/PC transplantation for chronic SCI, we used thoracic SCI mouse models to compare manifestations secondary to both transplantation and treadmill training, and the two therapies combined, with a control group. Significant locomotor recovery in comparison with the control group was only achieved in the combined therapy group. Further investigation revealed that NS/PC transplantation improved spinal conductivity and central pattern generator activity, and that treadmill training promoted the appropriate inhibitory motor control. The combined therapy enhanced these independent effects of each single therapy, and facilitated neuronal differentiation of transplanted cells and maturation of central pattern generator activity synergistically. Our data suggest that rehabilitative treatment represents a therapeutic option for locomotor recovery after NS/PC transplantation, even in chronic SCI.

Patients with spinal cord injury (SCI) experience various sequelae, such as motor paresis and spasticity, sensory disturbances, and bowel and rectal dysfunction. Although the injured central nervous system, including the spinal cord, shows only a small degree of plasticity, many studies on neural stem/progenitor cell (NS/PC) transplantation in the acute and subacute phase have shown significant recovery in locomotor and sensory function^1^,^2^. The results of these studies collectively suggest that the critical time window for transplantation therapy in rodents is around 7–10 days post-injury (DPI). Most studies targeting chronically injured spinal cord have reported no significant recovery of function^3^,^4^,^5^,^6^,^7^; however, there have been two exceptions to date^8^,^9^. In both of these exceptive studies, the cell transplantation was performed relatively early, in the “early chronic phase” at around 21 or 30 DPI^8^,^9^. This may indicate that the therapeutic window for transplantation closes by the end of the subacute phase or at the beginning of the early chronic phase. The majority of SCI patients are in the chronic phase, representing a major challenge for the clinical application of cell transplantation.

Recently, combined therapies for the treatment of chronically injured spinal cord have attracted the attention of researchers in regenerative medicine. One approach seeks to improve the viability or differentiation of NS/PC through the use of exogenous neurotrophic factors^5^. Another aims to degrade glial scarring or inhibit axonal growth inhibitors within the scar, by the use of chondroitinase ABC^10^,^11^ or semaphorin 3A inhibitor^12^,^13^. It has also been reported that combination therapy with a neurotrophic factor, Neurotrophin-3 expressing NS/PCs and chondroitinase ABC, led to superior functional recovery^4^. Although the potential importance of combination therapies involving cell transplantation and rehabilitation is widely recognised, there have been very few studies to date, all of which were limited to the acute and subacute phase of SCI^14^,^15^.

Treadmill training for SCI rodents is more established for rat models^12^,^16^,^17^. Rats have certain advantages over mice, which include their calmer temperament, greater endurance, and larger body size, which allows for easier handling. Although training methods for mice are not as well established, methods including wheel running^18^ and bipedal^19^ or quadrupedal^20^ treadmills have been introduced in a small number of trials conducted by a few research groups. Most studies of NS/PC transplantation have been performed using mice because of their amenability to genetic manipulation and the availability of bioluminescence imaging (BLI) for transplanted cells. The lack of an optimal well-suited model for both training and cell transplantation may be one reason why there have been very few studies of combination therapies and none in a chronic SCI animal model. In this study, we investigated how combination therapy with NS/PC transplantation and treadmill training affects behavioural function and histological manifestations in chronic SCI mice.

## Results

### Effects of treadmill training on survival rates and phenotype differentiation of grafted NS/PCs

**Figure 1:**
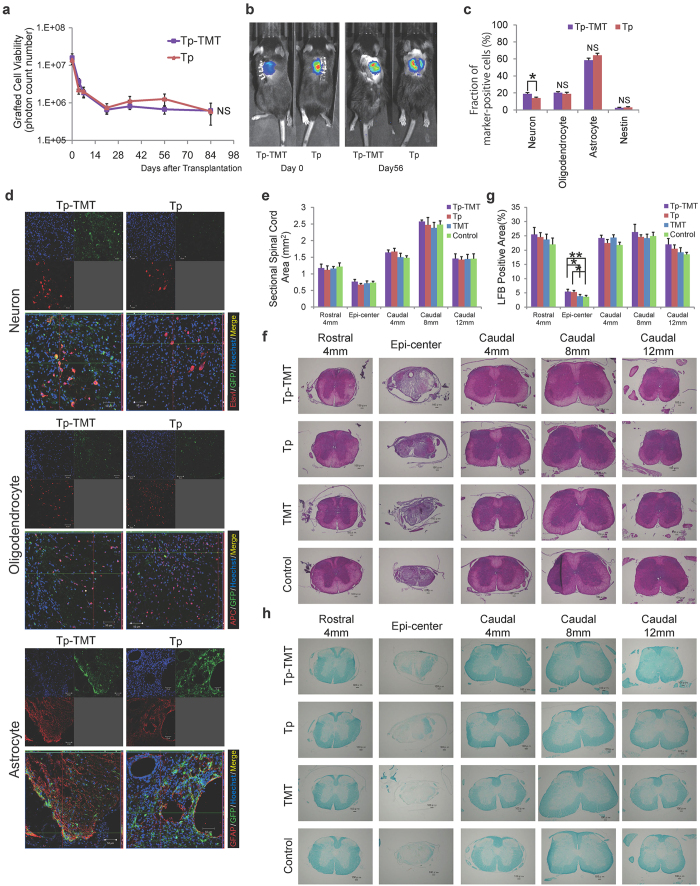
The effects of interventions for neural stem/progenitor cells. (**a**) Quantitative analysis using bioluminescence imaging (BLI) revealed that the survival rates of grafted cells were comparable between the Tp and Tp-TMT groups up to 84 days post-transplantation (the vertical axis has a logarithmic scale). Statistical analysis was performed using 2-way repeated measures ANOVA (Tp-TMT: *n* = 18, Tp: *n* = 19). (**b**) Representative BLI of animals in both groups. (**c**) The differentiation rate of grafted cells into the three neural cell lineages at 84 days post-transplantation. The proportion of Elavl+ cells is significantly higher in the Tp-TMT group than in the Tp group. No significant difference was observed in GFAP+ and APC+ cells. Only a small number of nestin + immature neural progenitor cells were observed in both groups. Statistical analysis was performed using paired *T*-tests (*n* = 6). (**d**) GFP positive grafted cells differentiated into Elavl+ neurons, GFAP+ astrocytes, and APC+ oligodendrocytes in Tp and Tp-TMT groups at the caudal site of the lesion (from the lower part of T9 to T10 level). Scale bar: 50 μm. (**e**) The sectional spinal cord area at each spinal level, which was similar in all four groups assayed. Statistical analysis was performed using the Tukey-Kramer test (*n* = 6). (**g**) The myelinated area at each spinal level, which was significantly larger in the two transplanted groups (Tp and Tp-TMT groups) than in the two non-transplanted groups (TMT and Control groups). Statistical analysis was performed using the Tukey-Kramer test (*n* = 6). (**f,h**) Representative HE- and LFB-stained images of axial sections at each level in each group. Images of each group were obtained from identical animals. Scale bars: 100 μm. *P < 0.05, **P < 0.01. Values are means ± SEM.

To investigate whether treadmill training changed the transplanted cell viability, BLI analysis was performed at 4, 7, 21, 35, 56, and 84 days post-transplantation. At 84 days post-transplantation, approximately 4% of the cells survived in both the combination transplantation and treadmill training (Tp-TMT) and the transplantation single therapy (Tp) groups (3.93 ± 2.33% vs. 4.25 ± 0.80%). The survival rates of the transplanted cells were comparable between the Tp-TMT group and the Tp only group (2-way repeated measures ANOVA; *P* = 0.855, Fig. 1a,b). Moreover, point-to-point analyses revealed no significant differences at each examined time point (*P* < 0.05, paired *T*-test).

To evaluate the differentiation phenotype of the grafted cells, immunohistochemical analyses for specific markers for each cell type were performed at 84 days post-transplantation for the Tp-TMT and the Tp only groups at the lesion epicentre and rostral and caudal sites. The following lineage specific markers were used; Elav-like (Elavl) for neurons, adenomatous polyposis coli antigen (APC) for oligodendrocytes, and glial fibrillary acidic protein (GFAP) for astrocytes. Quantitative analyses revealed that the proportion of Elavl+ neurons was significantly higher in the Tp-TMT group than in the Tp group (Tp-TMT: 18.91%, Tp: 14.02%, *P* = 0.0315, paired *T*-test), whereas no significant inter-group difference was observed for APC + oligodendrocytes (Tp-TMT: 21.32 ± 1.44%, Tp: 20.75 ± 2.69%, *P* = 0.855) and GFAP+ astrocytes (Tp-TMT: 57.21 ± 2.61%, Tp: 62.31 ± 2.59%, *P* = 0.195). Nestin-positive immature cells represented around 2.5–3.0% of the grafted cells in both groups (*P* = 0.799; Fig. 1c,d).

To evaluate the effects of NS/PC transplantation and/or training on the sectional spinal cord area and myelinated area, in all the experimental groups, axial sections at the lesion epicentre, and 4 mm rostral and 4, 8, and 12 mm caudal to it, were assessed histologically with haematoxylin-eosin (HE) and Luxol Fast Blue (LFB) staining. Although LFB does not directly label the myelin (LFB cannot distinguish between myelin debris-filled macrophages, myelinated axons, remyelinated axons, or partially demyelinated axons), it is often used as a reliable index to reflect the overall myelination within the injured spinal cord^5^,^7^,^21^,^22^. The experimental transverse area of the spinal cord did not significantly differ between any pair of groups, at any levels assessed, regardless of the transplantation, treadmill training, or combination of therapies (Fig. 1e,f). The LFB+ area at the epicentre was significantly higher in the two transplanted groups (Tp-TMT and Tp groups) than in the two phosphate-buffered saline (PBS) injected groups (TMT and Control groups; Tp-TMT: 5.4%, Tp: 5.3%, TMT: 3.8%, Control: 3.6%, *P* = 0.048, Tukey-Kramer test), whereas no significant difference was observed in the distant areas. There was no significant change induced by the TMT only therapy (Fig. 1g,h).

### Effect of transplantation and treadmill training on the fibres in the epicentre and lumbar enlargement

**Figure 2:**
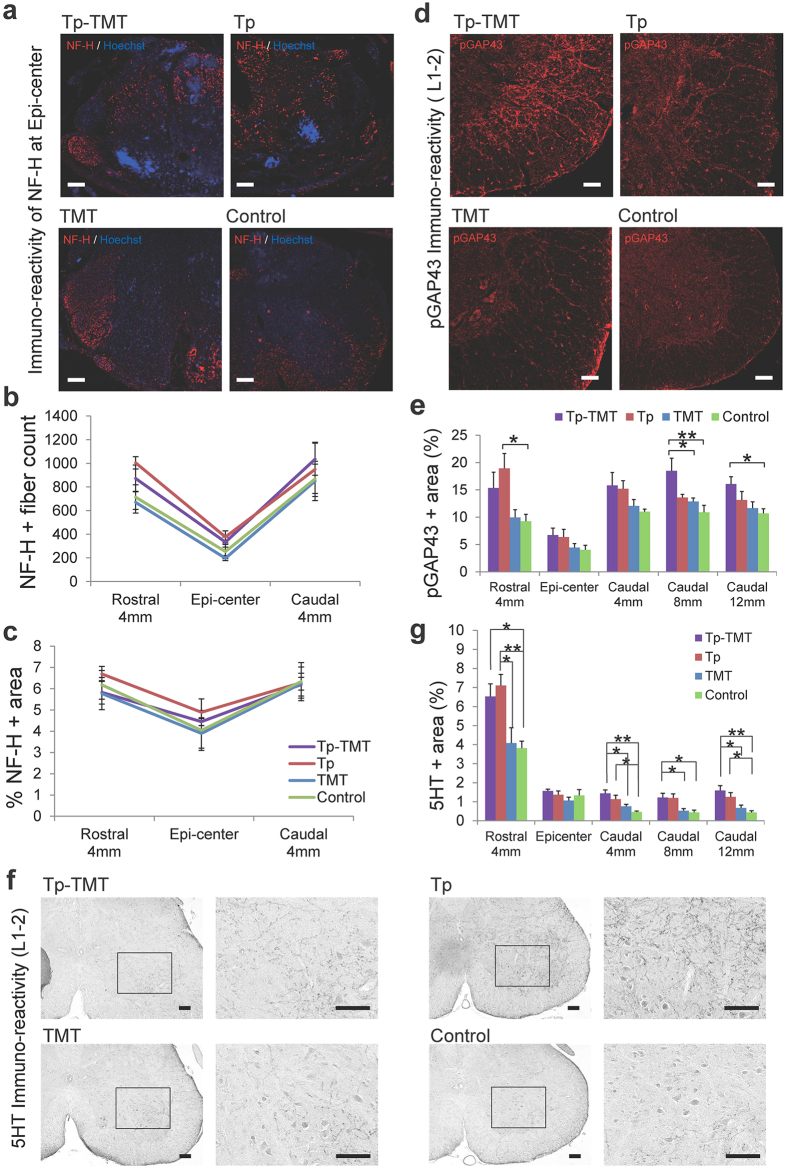
Fibres running through the lesion epicentre and lumbar enlargement. (**a**) Representative images of axial sections stained for NF-H around the lesion epicentre. (**b,c**) NF-H quantified with an NF-H+ dot count (**b**) and NF-H+ area (**c**). The values around the lesion epicentre were similar across the four groups for both assays. (**d**) Representative images of axial sections stained for pGAP43 at the lumbar enlargement. (**e**) Quantification of pGAP43+ area at the lesion epicentre and 4 mm rostral and 4, 8, and 12 mm caudal to the lesion across the four groups. Values increased in the two transplanted groups rostral to the lesion and were significantly greater only in the Tp-TMT group caudal to the lesion. (**f**) Representative images of axial sections stained for 5HT. (**g**) The areas of 5HT+ serotonergic fibres were significantly larger in the two transplanted groups, both rostral and caudal to the lesion epicentre, except for the epicentre itself. Statistical analyses were performed using the Tukey-Kramer test. Values are means ± SEM (*n* = 6). *P < 0.05, **P < 0.01. Scale bars: 100 μm in (**a,d,f**).

To quantify the fibres passing through the lesion related to the locomotor recovery, fibres positive to 200 kDa neurofilament (NF-H) were evaluated at the levels of the lesion epicentre, and 4 mm rostral and caudal to it, within all the experimental groups. Although it is known that increases in NF-H fibres occur secondary to subacute transplantation therapy^23^, the NF-H positive areas were comparable within all four groups, at all tested levels within the chronically injured spinal cord (*P* > 0.05, Tukey-Kramer test, Fig. 2a–c). The regenerative fibres were further assayed using immunoreactivity of phosphorylated Growth Associated Protein-43 (pGAP43), which is specifically localised to regenerating, but not intact, axons^24^. These assays were performed at the lesion epicentre and 4 mm rostral and 4, 8, and 12 mm caudal to it. In the transplanted groups, in sections 4 mm rostral and 4 mm caudal to the lesion, the pGAP43-positive area was significantly greater than in the non-transplanted groups. Although the increase of pGAP43 positive area in the Tp alone group was limited to the NS/PCs injected site, in the treadmill training combined group it was significantly greater than in the two non-transplanted groups, even in the lumbar enlargement distant from the epicentre (Tp vs Control: *P* < 0.05 at 4 mm rostral to epicentre; Tp-TMT vs TMT: *P* < 0.05 at 8 mm caudal to epicentre; Tp-TMT vs Control: *P* < 0.01 at 8 mm caudal to epicentre; *P* < 0.05 at 12 mm caudal to epicentre; Tukey-Kramer test; Fig. 2d,e).

To investigate the effect of interventions on the serotonergic activity, which is also known to promote locomotor recovery after SCI through activation of the central pattern generator (CPG)^25^,^26^, 5-hydroxytryptamine (5HT) positive fibres were immunohistologically assessed at the levels of the lesion epicentre, and 4 mm rostral and 4, 8, and 12 mm caudal to it. In the two transplanted groups, these fibres were significantly higher than in the two PBS injected groups, at all the levels assessed, except for the lesion epicentre. No significant effect was induced by the additional treatment with treadmill training (Tukey-Kramer test; Fig. 2f,g).

### Effects of transplantation and treadmill training on the neural circuit at the lumbar enlargement

**Figure 3:**
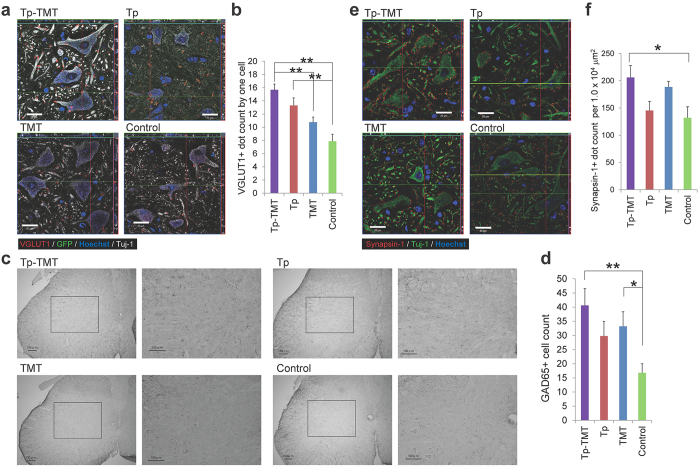
Effects of interventions on the neural circuit at the lumbar enlargement. (**a**) Representative images of axial sections stained for VGLUT1 at the anterior horn of the lumbar enlargement. (**b**) VGLUT1+ boutons are significantly increased in the two transplanted groups. Statistical analysis was performed using the Tukey-Kramer test. (**c**) Representative images of axial sections stained for GAD65 at the lumbar enlargement. (**d**) The number of GAD65+ cells is significantly higher in the Tp-TMT group than in the Control group. Statistical analysis was performed using the Tukey-Kramer test. (**e**) Representative images of axial sections stained for Synapsin-I at the anterior horn of the lumbar enlargement. (**f**) Synapsin-I+ boutons are significantly more numerous in the Tp-TMT group than in the Control group. Statistical analysis was performed using the Tukey-Kramer test. Values are means ± SEM (*n* = 6). *P < 0.05, **P < 0.01. Scale bars: 20 μm in (**a,e**), and 50 μm in (**c**).

To evaluate the effects of NS/PCs transplantation and treadmill training on the CPG in the lumbar enlargement, molecules related to both its excitatory and inhibitory control were assessed immunohistologically. In rodents, the CPG elements are distributed throughout the entire lumbar region, although neurons located in L1 and L2 are shown to have a higher rhythmogenic capability^27^,^28^,^29^. To investigate the excitatory activity in the lumbar enlargement, vesicular glutamate transporter 1 (VGLUT1) immunoreactive boutons around the motoneurons were quantified. These are known to provide an excitatory drive to the CPG^30^,^31^. Although no significant difference was observed in the number of VGLUT1+ boutons between Tp-TMT and Tp groups (*P* > 0.05), their numbers were significantly increased in both Tp-TMT and Tp groups in comparison with the controls (*P* < 0.01, Tukey-Kramer test; Fig. 3a,b), indicating that transplantation had a positive effect on the excitatory drives to the CPG.

For improved gait performance, appropriate inhibitory control of the gait pattern and excitatory drive are indispensable. The number of glutamate decarboxylase-65 (GAD65) positive neurons was therefore quantified in lamina V–VII of the lumbar enlargement. These neurons are known to decrease in number after SCI, and are also known to be related to the rhythmic-coordinative inhibition of the gait and the manifestation of behavioural spasticity^16^,^32^,^33^,^34^. Although no significant difference was observed in the number of GAD65+ neurons between the Tp-TMT and Tp groups, the number was significantly higher in the Tp-TMT group than in the Control group (*P* < 0.05, Tukey-Kramer test; Fig. 3c,d), indicating that the combination of treadmill training with transplantation had an additional beneficial effect on coordinative control.

To further clarify the effects of the combination therapy on the maturation of the neural circuit, the number of synapses was further assessed according to the extent of Synapsin-I immuno-reactive boutons in the axial sections of the lumbar enlargement. Synapsin-1 is widely used as a presynaptic marker to examine activity-dependent synaptic plasticity and synaptic function^12^. It was significantly higher in the Tp-TMT group than in the Control group (*P* < 0.05, Tukey-Kramer test; Fig. 3e,f), indicating that a combination of NS/PC transplantation and treadmill training promoted the maturation of synapses in the chronic post-transplantation spinal cord.

### Functional recovery was enhanced by the combination of NS/PC transplantation and treadmill training

**Figure 4:**
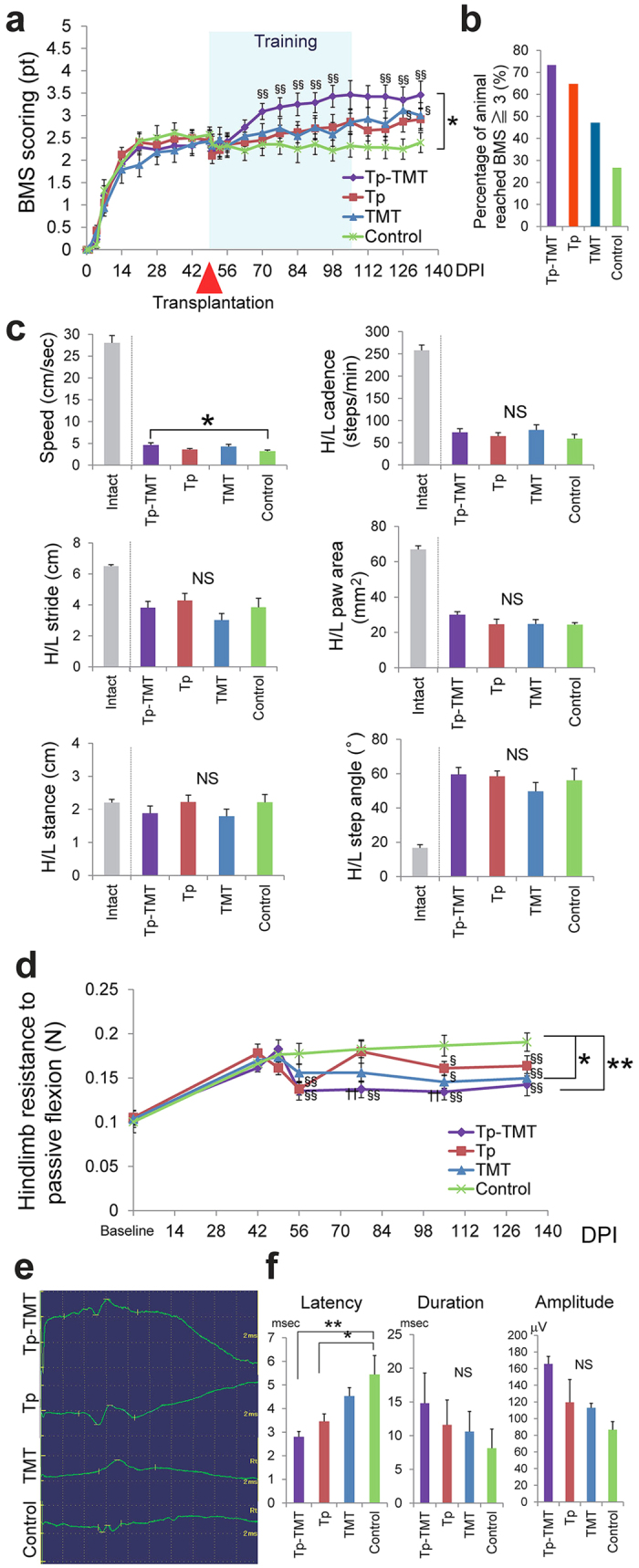
Behavioural and electrophysiological manifestations in each group. (**a**) The locomotor recovery represented by the Basso Mouse Score (BMS) in each of the four groups. The Tp-TMT group exhibited significantly better functional recovery than the control group. Statistical analyses were performed using repeated measures ANOVA followed by point to point comparisons (Tp-TMT: *n* = 18, Tp: *n* = 19, TMT: *n* = 17, Control: *n* = 16). *P < 0.05, in repeated measures ANOVA. ^§§^*P* < 0.01 and ^§^*P* < 0.05 compared to Control group in Tukey-Kramer tests. (**b**) The percentage of animals reaching a score of 3 or above in the BMS scoring. (**c**) Gait dynamics assessed as gait speed, hindlimb (H/L) step pitch, H/L stride length, H/L stance width, and H/L step angle in each of the four groups at 133 days post-injury (DPI). The values for intact mice are cited from previous studies^74^,^75^. Statistical analyses were performed using Tukey-Kramer tests (Tp-TMT: *n* = 15, Tp: *n* = 15, TMT: *n* = 12, Control: *n* = 11). *P < 0.05, NS: Not significant. (**d**) The transition over time of the resistance to flexing of animals’ hindlimbs, which were passively measured using a strain-gauge in each of the four groups. The spasticity in the Tp-TMT and TMT groups was significantly reduced compared with the Control group. Statistical analyses were performed using repeated measures ANOVA followed by point to point comparisons (Tp-TMT: *n* = 16, Tp: *n* = 15, TMT: *n* = 15, Control: *n* = 13). *P < 0.05 and **P < 0.01, in repeated measures ANOVA. ^§§^*P* < 0.01, ^§^*P* < 0.05 compared to Control group, and ƗƗ *P* < 0.01 compared to Tp group in Tukey-Kramer tests. (**e**) Representative MEPs in the four groups at 133 DPI. (**f**) The latency, duration, and amplitude of the MEP are shown. The latency was significantly shortened in Tp-TMT and Tp groups. Statistical analyses were performed using Tukey-Kramer tests (Tp-TMT: *n* = 10, Tp: *n* = 10, TMT: *n* = 10, Control: *n* = 9). *P < 0.05. Values are means ± SEM.

Locomotor recovery was behaviourally assessed with respect to open-field locomotor function, footprint gait analysis, and spasticity. The open-field locomotor function was assessed with Basso-Mouse-Scale (BMS) scoring up to 133 DPI. It was significantly improved in the Tp-TMT group compared with the Control group (repeated measures ANOVA, multiple comparison, *P* = 0.035), whereas no significant change was detected between the control group and each of the single therapies. Point-to-point comparisons further demonstrated a significant benefit of the combination therapy compared with each of the single therapies. In this assessment, significant differences between Tp and Control groups were also revealed at 126 and 133 DPI (Steel-Dwass multiple comparison tests; Fig. 4a). The percentage of animals reaching a score of 3 or above in the BMS scoring (which corresponds to weight-bearing stepping with the hindlimb or more) was 73.3% for Tp-TMT, 64.7% for Tp, 47.1% for TMT, and 26.7% for the Control group (Fig. 4b).

Gait performance was further analysed using the DigiGait system, which revealed gait speed in the Tp-TMT group to be significantly faster than in the Control group (*P* = 0.026). No other significant changes were observed in gait performance, as measured by step cadence, stride length, hindlimb paw area, hindlimb step angle, or hindlimb step width (Steel-Dwass multiple comparison tests, *P* > 0.05 for all; Fig. 4c).

Hindlimb spasticity was assessed as the resistance to full flexion from full extension using strain-gauge testing. Spasticity was significantly suppressed in the Tp-TMT and TMT groups relative to the Control group (Fig. 4d; Tp-TMT vs Control: *P* < 0.01, TMT vs Control: *P* < 0.05, repeated measures ANOVA multiple comparison). Whereas significant increases in the spasticity were observed between the baseline time point and the last time point in the Tp only (baseline: 0.105 ± 0.007 vs 133 DPI: 0.164 ± 0.011, *P* < 0.01), TMT only (baseline: 0.103 ± 0.009 vs 133 DPI: 0.150 ± 0.009, *P* < 0.01), and Control groups (baseline: 0.100 ± 0.012 vs 133 DPI: 0.190 ± 0.010, *P* < 0.01), no significant difference was observed in the Tp-TMT group (baseline: 0.105 ± 0.008 vs 133 DPI: 0.142 ± 0.012, *P* = 0.089). A point-to-point comparison revealed that spasticity in the two transplanted groups was significantly suppressed in the early phase after transplantation therapy compared with the Control group. In the late phase, spasticity was significantly suppressed in each of the single therapies and the combination therapy, in comparison with the Control group (Fig. 4d; Tukey-Kramer tests).

The motor evoked potential (MEP) was assessed to clarify the effect of interventions on the spinal cord conduction capability. The latency of the evoked compound motor action potential was significantly shorter in the two transplanted groups than in the Control group, whereas no significant difference was induced by the addition of treadmill training (Tp-TMT vs TMT: *P* < 0.05, Tp-TMT vs Control: *P* < 0.01, Tp vs Control: *P* < 0.05, Tukey-Kramer test). Both the duration and the amplitude of the MEP were comparable between the four groups (*P* > 0.05; Fig. 4e,f).

## Discussion

In this study, we show for the first time that a combination therapy with NS/PC transplantation and treadmill training can promote functional recovery, even in chronic SCI animal models. Although a paradigm for meaningful recovery has been established, which requires therapy combining neurotrophic factors, suppression of axonal growth inhibitors, rehabilitation toward functional reorganisation, and regenerative cell replacement therapy^4^,^5^,^7^, to our knowledge there have been no reports on the effects of a combined therapy with treadmill training and transplantation in chronic animal models.

In the present study, no significant locomotor improvement was detected in the Tp only or TMT only groups, in comparison with the Control group. However, the following results indicate that each single therapy promoted potentially beneficial changes in chronically injured spinal cord. Firstly, NS/PC transplantation enhanced electrophysiological recovery, such as the shortening of MEP latency, compared with the Control group (Fig. 4e,f), which is consistent with the histological finding of an increase in the area of LFB myelin labelling at the lesion epicentre (Fig. 1g,h). Although there is controversy over whether re-myelination enhancement is secondary to cell therapy in chronic SCI, this finding is compatible with those of previous reports^35^,^36^. Secondly, our results indicate that NS/PC transplantation up-regulates CPG activity, which is supported by the histological findings that 5-HT positive fibres (Fig. 2f,g) and VGLUT1 positive boutons around the motoneuron (Fig. 3a,b) are significantly increased in the transplanted groups. This reflects the excitatory activity of the CPG^25^,^26^,^30^,^31^.

**Figure 5:**
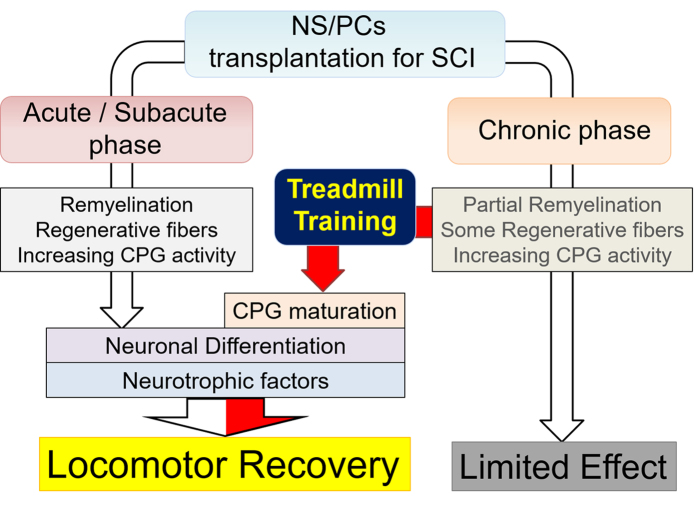
A scheme summarizing the beneficial mechanisms brought about by each intervention. The effects of neural stem/progenitor cell transplantation and treadmill training therapies towards functional recovery in the chronic and acute/subacute SCI model animals. Although transplantation single therapy induces only a limited effect on partial remyelination, 5HT fibre regeneration, and increasing CPG activity without appropriate inhibition, the addition of treadmill training further facilitates neuronal differentiation and CPG maturation, together with trophic support, leading to significant locomotor recovery.

Treadmill training facilitates suppressive regulation related to coordinated rhythmic motor control within the lumbar enlargement, even in the chronically injured spinal cord, as shown in previous studies on subacute SCI^16^,^37^. This is supported by the finding that treadmill training improved behavioural manifestations of spasticity (Fig. 4d) and increased GABAergic activity within the lumbar enlargement (Fig. 3c,d). Furthermore, neuronal differentiation and the phenomenon of appropriate inhibition recovery and/or synaptic regeneration, which we have termed “CPG maturation”, are promoted by the addition of rehabilitative therapy. The case for CPG maturation is supported by the finding of increased pGAP43 immunoreactivity (Fig. 2d,e) and synapsin-I+ synapse numbers (Fig. 3e,f). In Fig. 5 and Table 1, we have summarised the mechanisms contributing towards functional recovery that were brought about by either the transplantation or treadmill training therapies separately, or by their synergistic effects. However, it is notable that these observations provide only limited support for each aspect of recovery; this study outlines only the profiles of recovery secondary to the combination therapy of transplantation and treadmill training. Therefore, further investigations on each aspect of the findings are needed for verification.

Trophic support is one of the key factors behind the effect of combination therapy; both NS/PC transplantation and treadmill training have the potential to supply neurotrophic factors to the injured spinal cord, and the effects of combined NS/PC transplantation and treadmill training are facilitated by the combined secretion of more neurotrophic factors than in the single conditions. It is known that various neurotrophic factors are up-regulated in subacute transplantation models^23^,^38^,^39^, and this up-regulation is even maintained in chronically injured spinal cord^6^. When neurotrophic factors are combined with treadmill training, functional recovery is enhanced, regardless of the phase after injury^40^,^41^,^42^,^43^. Similarly, treadmill training up-regulates various neurotrophic factors in both subacute and chronic SCI^15^,^17^,^44^,^45^,^46^. Previous studies have demonstrated that the combination of NGF or IGF-1 with NS/PC transplantation exerts a neuroprotective effect^5^,^47^. BDNF and NGF promote neuronal differentiation^48^,^49^, and BDNF and NT-3 both enhance locomotor functional recovery in subacute SCI^50^,^51^,^52^,^53^. Furthermore, transplantation of NS/PCs expressing NT-3 induces significant locomotor recovery, even after chronic SCI^5^. Taken together, we suggest that such trophic support enhances the intrinsic ability of these interventions for inducing locomotor recovery.

The refractory state of chronically injured spinal cord interferes with the functional recovery secondary to NS/PC transplantation^6^,^7^. We suggest that the following rehabilitation-specific effects may contribute to the functional recovery observed in our study: 1) activity-dependent neuronal plasticity and modification of neural circuit(s), and 2) the treatment of learned non-use. Both task-specific and use-dependent neuronal plasticity and neural circuit modification involve the formation of new neuronal circuits^54^, reinforcement of locomotor networks in a more selective and stable manner^55^, changes in the synapse strength with long-term structural change^56^, limiting maladaptive plasticity through training^57^, spinal fixation through the peripheral sensory input from the use of paretic limbs^58^, and reorganisation of the cortical network^59^. We consider the principle of our rehabilitative intervention, in which we encouraged the voluntary gait, should have resulted in specific sensorimotor input into the lumbar spinal cord. This would be suitable for promoting these beneficial effects. Researchers have reported that specific training paradigms to encourage voluntary stepping are more effective than completely passive stepping^16^,^60^. Instrumental training promotes activity-based learning and suppresses maladaptive plasticity^57^, whereas inappropriate training intervention can reduce the capacity for motor learning^61^. These plasticity changes in the circumstances where NS/PCs were transplanted and survived may affect their fate and function. Furthermore, in the chronic SCI animals, “learned non-use”^62^ and disuse may trigger dysfunction, which could further suppress the impaired hindlimb activity, thereby masking the beneficial effects of transplantation. Animals in the Tp group consistently showed a gradual functional recovery, and even a significant difference compared with the control animals, when given pre-training before NS/PC transplantation in this study, as distinct from previous studies on chronic SCI transplantation^3^,^4^,^5^,^6^,^7^. This concept would further support the idea that even one week of voluntary or instrumental training can induce the up-regulation of significant amounts of neurotrophic factors like BDNF, which promote spinal learning^42^,^63^. Thus, even short-term conditioning training before NS/PC transplantation may induce beneficial changes by partly releasing animals from undesirable behavioural states.

No significant difference was observed in locomotor function between the Tp-TMT and TMT groups. Although the recovery in the Tp-TMT group was significant compared with the Control group, this does not necessarily indicate that combination therapy is effective for chronic SCI. Our combination therapy was not sufficiently effective to repair the lesion epicentre, as there were no significant changes in residual spinal volume, spared fibres, 5HT-positive fibres, regenerative fibres, or synaptic generation. Previous studies have shown that glial scarring composed of chondroitin-sulfate-proteoglycans physically impedes regenerative activity, and chemically impedes it by the generation of various effectors, such as semaphorin 3A. This is especially the case in chronically injured spinal cord^13^. In this regard, many researchers have investigated the efficacy of combinatorial drug treatments for inhibition of these factors in chronic SCI^4^,^11^,^64^. Further combined treatments targeting glial scarring and/or axonal growth inhibitors using chondroitinase ABC or semaphorin3A inhibitors in combination with NS/PC transplantation and rehabilitation represent a promising future strategy.

In conclusion, rehabilitative treatments represent a third therapeutic option to facilitate locomotor recovery after NS/PC transplantation. Together with the effect of transplantation itself, treadmill training promotes functional recovery through neuronal differentiation and CPG maturation, even in chronic SCI animal models. Comprehensive treatment, including cell replacement, medication, and rehabilitation, may therefore be useful in the treatment of chronically injured spinal cord, refractory to each of these interventions alone.

## Materials and Methods

### Study approval

All experiments were approved by the Animal Ethics Committee of Keio University (Tokyo, Japan; No. 12082-1) and were performed in full compliance with the Guide for the Care and Use of Laboratory Animals (National Institutes of Health, Bethesda, MD).

### Experimental design

**Figure 6:**
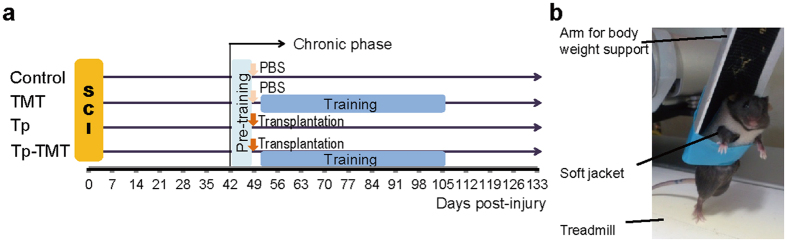
Experimental design and training device. (**a**) The complete experimental schedule. With the beginning of the chronic phase (42 DPI), all of the experimental animals are pre-trained for 1 week. Neural stem/progenitor cell transplantation or PBS injection is performed 49 days post-injury, and is then followed by 8 weeks of training period. (**b**) Mice are trained with a partial body weight supported bipedal gait using a commercially available robotic device.

To investigate the profiles of the change induced by each of the treadmill training and transplantation therapies, a four-armed design with groups composed of combination therapy (Tp-TMT) transplantation single therapy (Tp), treadmill training single therapy (TMT), and a control group (Control) was applied. As shown in Fig. 6, the NS/PCs were transplanted at 49 DPI. Treadmill training was performed for two independent periods following different strategies: pre-training for all of the SCI mice at 42–48 DPI, and intervention training for mice in the TMT and Tp-TMT groups at 52–105 DPI (Fig. 6a).

### Animals

Eighty C57BL/6J mice (8–9 weeks old, female, 18–22 g; Clea, Tokyo, Japan) were used in this study. Because some animals died in the process of the experiment, a total of seventy were included in the results. The sample size for each experiment was determined according to averages and standard deviations were calculated from preliminary experiments. The animals were housed 3–5 per cage in an accredited facility. The animals were maintained on a 12 h light/dark cycle with access to food and water ad libitum.

### NS/PC culture

NS/PCs were cultured and expanded as previously described^65^. The source of NS/PCs was selected according to previous reports^1^,^66^,^67^. Briefly, the cells were harvested from the striata of transgenic mice established from C57BL/6J on embryonic day 14. These mice ubiquitously express ffLuc-cp156, which is a fusion protein of a yellow variant of Aequorea GFP and firefly luciferase^51^. Dissociated cells were collected and re-suspended in culture medium composed of Dulbecco’s modified Eagle medium/F12 (Sigma-Aldrich, St. Louis, MO, USA) with hormone mixture. Human recombinant FGF-2 (Peprotech, Rocky hill, NJ, USA) and EGF (Peprotech; 20 ng/ml each) were added every 2 days. Cells were expanded for three passages and the neurospheres were used for cell transplantation.

### SCI model and grouping

Severe lower thoracic level contusive SCI was performed as described previously^7^. Briefly, all mice were anesthetised with an intraperitoneal (i.p.) injection of ketamine (100 mg/kg) and xylazine (10 mg/kg). Following T9 laminectomy, a 70 kilodyne contusive injury was applied to the exposed dura mater using a commercially available SCI device (IH Impactor, Precision Systems and Instrumentation, Lexington, KY, USA). For 3 days after the injury, 12.5 mg/kg ampicillin was administered intramuscularly. All injured mice received twice-daily manual bladder evacuations until recovery of function. The SCI animals were randomly assigned to each group (20 mice per group). As some mice died in the process of the interventions, the following numbers of animals were included in the analyses: Tp-TMT, *n* = 18; Tp, *n* = 19; TMT, *n* = 17; and Control, *n* = 16. All the information regarding the groups was handled separately and investigators performing behavioural assessments were blind to this information.

### NS/PC transplantation

NS/PCs (approximately 5 × 10^5^ cells/2 μl) were transplanted separately into regions 1 mm rostral and caudal to the rim of the lesion epicentre at 49 DPI, following the method reported in a previous study^68^. In brief, NS/PCs were injected with a glass micropipette at a rate of 1 μl/min using a Hamilton syringe (25 μl, Hamilton, Bonaduz, Switzerland) and a stereotaxic microinjector (KDS 310, Muromachi-kikai Co. Ltd., Tokyo, Japan). In the TMT and Control groups, PBS was injected in the same manner, instead of the NS/PCs.

### Bioluminescence imaging

*In vivo* BLI analysis was performed with a Xenogeny-IVIS spectrum CCD optical macroscopic imaging system (Caliper LifeSciences, Hopkinton, MA, USA) as previously reported^1^,^7^,^68^. Briefly, the signal intensity from the transplanted cells was recorded as the maximum 5 min integration of bioluminescence over the period from 15 to 45 min post i.p. injection of D-luciferin (0.3 mg/g). Images were analysed with Living Image software (Caliper LifeSciences), and the signal intensity was measured as photon flux.

### Treadmill training

SCI animals underwent partial body weight supported, voluntary bipedal gait training using a commercially available treadmill training device (Rodent Robot 3000; Robomedica Inc., Irvine, CA; Fig. 6b)^16^. The training intervention was performed for two purposes in two different periods; the conditioning training was conducted for all animals before the injection of NS/PCs or PBS from 42 to 48 DPI, and the intervention training was conducted post-injection for animals in TMT and Tp-TMT groups from 52 to 105 DPI. The training was performed for 20 minutes per day, 5 days per week, with approximately 80% to 90% of the animals’ body weight supported. The speed of the treadmill was set between 0.5 to 1.5 cm/s. Weight-support and speed were adjusted on every intervention day to optimally induce a voluntary gait.

### Behavioural analyses

Hindlimb locomotor function was evaluated weekly up to 133 DPI using the Basso-Mouse-Scale (BMS)^69^. Quadrupedal gait dynamics regarding gait speed, cadence, stride length, paw area, stance width, and step angle of hindlimbs were evaluated from the footprints of the mice using a DigiGait imaging and analysis system (Mouse Specifics, Boston, MA)^21^,^70^. Hindlimb spasticity was assessed as the resistance to full flexion from full extension^71^. The maximum force required was measured using a handheld strain-gauge (model FGP-0.5; Nidec-Shimpo, Kyoto, Japan) and the average value of six measurements was recorded^16^.

### Electrophysiology

MEPs were recorded using a Neuropack S1 MEB-9402 (Nihon Kohden, Tokyo, Japan) at 133 DPI, as previously described^21^. Briefly, a C1 laminectomy was performed after anesthetisation with an i.p. injection of ketamine (60 mg/kg) and xylazine (6 mg/kg). Stimulation was applied from the C0 level of the spinal cord with a wired electrode, and the MEP was picked up from the quadriceps muscle of the hindlimbs by a needle electrode. The intensity of the stimulus was set to trigger a supra-maximum response (approximately 1.0–2.0 mA intensity), and the duration and the stimulus frequency were set to 0.2 ms and 1 Hz, respectively. The latency, amplitude, and duration of the induced potential were recorded.

### Histological analyses

At 133 DPI, anesthetised animals were transcardially perfused with PBS, followed by fixation with 4% paraformaldehyde (in 0.1 M PBS (*n* = 6), each group). The spinal cords were removed, postfixed overnight in 4% paraformaldehyde, soaked overnight in 10% sucrose, followed by 30% sucrose, embedded in Optimal Cutting Temperature compound (Sakura Finetechnical Co., Ltd., Tokyo, Japan), and frozen as previously described^7^. A cryostat (CM3050 S; Leica Microsystems, Wetzlar, Germany) was used to dissect sections (20 μm thick) out of the injured spinal cords from 4 mm rostral to the lesion epicentre to 12 mm caudal to the lesion epicentre. The sections of transplanted spinal cord were then subjected to histological analyses. For assessment of NS/PC differentiation, and quantification of NF-H, pGAP43, VGLUT1, Synapsin-I, 5HT, and GAD65, the sections were incubated at 4 °C overnight with primary antibodies, and then incubated with appropriate secondary antibodies after washing. Supplementary Table 1 lists the primary antibodies used in the study. Nuclei were stained with Hoechst 33258 (10 μg/ml, Sigma-Aldrich). All images except for HE, LFB, 5HT and GAD65 were obtained using a confocal laser scanning microscope (LSM 780; Carl Zeiss, Munich, Germany). Images of HE, LFB, 5HT and GAD65 staining were captured using fluorescence microscopy (BZ-9000; Keyence, Tokyo, Japan).Immunoreactivity of 5HT and GAD65 was analysed using 3,3′-diaminobenzidine immunohistochemistry. A biotinylated secondary antibody (Jackson ImmunoResearch Laboratories Inc., West Grove, PA, USA) was used after exposing the sections to 0.3% H_2_O_2_ to inactivate endogenous peroxidases. Signals were enhanced with the Vectastain ABC kit (Vector Laboratories, Inc., Burlingame, CA, USA). Diaminobenzidine (Wako; 0.005%) was used as a chromogen, and the reactions with 0.0075% hydrogen peroxide in water with Tris buffer were sustained for 3 min. Threshold values were maintained at constant levels for all analyses.

### Quantitative analyses

To quantify the proportion of each differentiated cell phenotype among the *in vivo* grafted cells, five regions were captured within axial sections at 200× magnification. GFP and phenotypic marker double-positive cells were counted in five sections, for six animals per group, at the lesion epicentre and at the rostral and caudal sites (T8–T10 level), as previously described^7^,^68^. The sectional spinal area was determined using HE- and LFB- stained images of axial sections from the lesion epicentre and 4.0 mm rostral and 4.0, 8.0, and 12.0 mm caudal to the epicentre, captured at 100x magnification (*n* = 6, each group). The myelinated areas were measured as for LFB+, in the manner following previous studies^7^,^68^,^72^. The NF-H+ fibres were quantified using axial sections from the lesion epicentre and 4.0 mm rostral and 4.0 mm caudal to the epicentre, captured at 200x magnification. The whole axial sections were evaluated for immunoreactive area and the number of fibres running through the section. The pGAP43+ and the 5HT+ fibres were evaluated using axial sections from the lesion epicentre and 4.0 mm rostral and 4.0, 8.0 and 12.0 mm caudal to the epicentre, captured at 200x magnification. The pGAP43+ fibres were quantified for immunoreactive area within the whole axial section, and the 5HT+ fibres were quantified for immunoreactive area within the automatically captured images of spinal grey matter. ImageJ software was used for these analyses (version 1.47; National Institutes of Health, USA). The average value of the neighbouring three sections was recorded (*n* = 6, each group) following the methods of previous studies^7^,^12^,^26^,^68^. VGLUT1 and synapsin-1 immunoreactive synapses were assessed using the axial sections of the lumbar enlargement captured at 630x magnification, and following procedures described in previous studies (*n* = 6, each group)^12^,^30^,^31^. Briefly, the numbers of VGLUT1 or synapsin-1 immunoreactive boutons around a motoneuron labelled with Tuj-1 were counted from three optical sections for one cell. Twelve randomly selected motoneurons from six optical sections within lamina IX at L1–2 were assayed in this manner for one animal. GAD65 + cells were determined using the axial sections of the lumbar enlargement captured at 100x, according to the methods in previous studies (*n* = 6, each group)^33^,^34^. The average number of GAD65+ neurons within lamina V–VII was counted, and then the average value from three different levels at L1–2 of the lumbar enlargement was calculated. The threshold values were maintained at a constant level for all analyses.

### Statistical analysis

All data are presented as mean ± SEM. Two-way repeated measures ANOVAs, along with point-by-point comparisons with Steel-Dwass tests or Tukey-Kramer tests, were used to examine the differences between groups in assessments of the BMS score, strain-gauge test, and grafted cell viability, following methods in a previous study^73^. The comparisons between the four groups were performed using Tukey-Kramer tests (*i.e.*, the parameters from the behavioural DigiGait and electrophysiological MEP analyses, histological assessments regarding sectional spinal area with HE staining, myelinated area with LFB staining, NF-H+ dot count and area, pGAP43+ area, 5-HT+ area, VGLUT1+ dot count, GAD65+ area, and synapsin-1+ dot count). The comparisons between two groups (*i.e.*, assessments on each lineage in the cell differentiation assay) were performed using *T*-tests.

## Additional Information

**How to cite this article**: Tashiro, S. *et al*. Functional Recovery from Neural Stem/Progenitor Cell Transplantation Combined with Treadmill Training in Mice with Chronic Spinal Cord Injury. *Sci. Rep.* **6**, 30898; doi: 10.1038/srep30898 (2016).

## Supplementary Material

Supplementary Information

Supplementary Video 1

Supplementary Video 2

## Figures and Tables

**Table 1:** Effects of each of the single therapies and the combination therapy on specific aspects of recovery that may lead to functional recovery.

	Tp	TMT	Tp-TMT
Locomotor function	**±**	**±**	**+**
Spasticity	**−**	**+**	**+**
Recovery within Lesion epicentre	**−**	**−**	**−**
Improvement in transplanted cell survival	**NA**	**NA**	**−**
Neuronal differentiation of transplanted cells	**+**	**NA**	**++**
Remyelination	**+**	**−**	**+**
Increasing excitability of CPG	**+**	**−**	**+**
Appropriate inhibition of CPG	**−**	**+**	**+**
Maturation of CPG	**−**	**−**	**+**
