# Supplementary material for: Functional Recovery from Neural Stem/Progenitor Cell Transplantation Combined with Treadmill Training in Mice with Chronic Spinal Cord Injury
Source: Sci Rep. 2016 Aug 3;6:30898. doi: 10.1038/srep30898 (PMC4971501; doi:10.1038/srep30898)
Supplement: Supplementary Information [file srep30898-s1.doc]

**Functional Recovery from Neural Stem/Progenitor Cells Transplantation Combined with Treadmill Training in Mice with Chronic Spinal Cord Injury**

Syoichi Tashiro^1^, Soraya Nishimura^2^, Hiroki Iwai^2^, Keiko Sugai^2^, Liang Zhang^1,3^, Munehisa Shinozaki^3,4^, Akio Iwanami^2^, Yoshiaki Toyama^2^, Meigen Liu^1^, Hideyuki Okano^3^ and Masaya Nakamura^2^

1. Department of Rehabilitation Medicine, Keio University School of Medicine, Tokyo, Japan
2. Department of Orthopaedic Surgery, Keio University School of Medicine, Tokyo, Japan
3. Department of Physiology, Keio University School of Medicine, Tokyo, Japan
4. Department of Neuroscience, City College of the City University of New York, NY, USA

**Corresponding Authors**

Prof. Masaya Nakamura, M.D., Ph.D., Department of Orthopaedic Surgery, Keio University School of Medicine, 35 Shinanomachi, Shinjuku, Tokyo, 160-8582 Japan. Tel.: +81-3-5363-3812; E-mail: masa@a8.keio.jp

Prof. Hideyuki Okano, M.D., Ph.D., Department of Physiology, Keio University School of Medicine, 35 Shinanomachi, Shinjuku, Tokyo, 160-8582 Japan. Tel.: +81-3-5363-3746; E-mail: hidokano@a2.keio.jp

**Author contributions**

ST, YT, ML, HO and MN designed the research; ST, SN and ZL conducted research; SN and KS provided NS/PCs, ST, HI and MS acquired data; ST and AI analyzed the data; ST, HO and MN wrote the manuscript; and MN and HO supervised all the experiments. All authors read and approved the final manuscript.

**Acknowledgments**

We appreciate the help of all the members of the spinal cord research team at the Department of Orthopaedic Surgery, Physiology, and Rehabilitation Medicine at Keio University’s School of Medicine. We also thank Dr. D. Sipp at RIKEN Center for Developmental Biology for the critical reading of the manuscript.

**Declaration of Conflicting Interests**

The author(s) declared the following potential conflicts of interest with respect to the research, authorship, and/or publication of this article: H. Okano is a scientific consultant for SanBio Co, Ltd (Japan); Eisai Co, Ltd (Japan); and Daiichi Sankyo Co, Ltd (Japan). The remaining authors report no conflicts of interest.

**Funding**

The author(s) disclosed receipt of the following financial support for the research, authorship, and/or publication of this article: This work was supported by grants from the following organizations: the Japan Science and Technology-California Institute for

Regenerative Medicine collaborative program; the Grants-in-Aid for Scientific Research from the Japan Society for the Promotion of Science (SPS) and the Ministry of Education, Culture, Sports, Science, and Technology of Japan (MEXT); This work was also supported by Research Center Network for Realization of Regenerative Medicine from by the Japan Science and Technology Agency (JST) and the Japan Agency for Medical Research and Development (AMED) (to H.O. and M.N.). H.O. is a paid scientific advisory board member for SanBio Co., Ltd.; the Keio Gijuku Academic by a Grant-in-Aid for Scientific Research on Innovative Areas (Comprehensive Brain Science Network) from MEXT; and the General Insurance Association of Japan.

**Supplementary Table. 1 Antibody list**

**Video legend.  Locomotion before and after the combined therapy**

Representative videos of the locomotion before and after intervention of a mouse in the Tp-TMT group are shown (42 DPI and 133 DPI). The locomotor function including hindlimb weight bearing and both left-right and forelimb-hindlimb coordination had improved after the intervention.
